# Gestation at completion of prenatal questionnaires in ALSPAC

**DOI:** 10.12688/wellcomeopenres.15976.2

**Published:** 2020-10-07

**Authors:** Yasmin Iles-Caven, Kate Northstone, Jean Golding

**Affiliations:** 1 Population Health Sciences, Bristol Medical School, University of Bristol, Oakfield House, Oakfield Grove, Bristol, BS8 2BN, UK

**Keywords:** ALSPAC, pregnancy, questionnaire administration, mothers, fathers, birth cohort study

## Abstract

Enrolling a cohort in pregnancy can be methodologically difficult in terms of structuring data collection. For example, some exposures of interest may be time-critical while other (often retrospective) data can be collected at any point during pregnancy.  The Avon Longitudinal Study of Parents and Children (ALSPAC) is a prime example of a cohort where certain data were collected at specific time points and others at variable times depending on the gestation at contact.

ALSPAC aimed to enrol as many pregnant women as possible in a geographically defined area with an expected date of delivery between April 1991 and December 1992. The ideal was to enrol women as early in pregnancy as possible, and to collect information, when possible, at two fixed gestational periods (18 and 32 weeks). A variety of methods were used to enrol participants.

Approximately 80% of eligible women resident in the study area were enrolled. Gestation at enrolment ranged from 4-41 (median = 14) weeks of pregnancy. Given this variation in gestation we describe the various decisions that were made in regard to the timing of questionnaires to ensure that appropriate data were obtained from the pregnant women.  45% of women provided data during the first trimester, this is less than ideal but reflects the fact that many women do not acknowledge their pregnancy until the first trimester is safely completed. Data collection from women at specific gestations (18 and 32 weeks) was much more successful (80-85%).

Unfortunately, it was difficult to obtain environmental data during the first trimester. Given the time critical nature of exposures during this trimester, researchers must take the gestational age at which environmental data was collected into account. This is particularly important for data collected using the questionnaire named ‘Your Environment’ (using data known as the A files).

## Introduction

There are considerable problems in child development that have been shown to be causally related to both genetic susceptibility and environmental impact. Three key examples that raised awareness of the importance of antenatal environmental exposures are: (i) the thalidomide tragedy, which resulted in unusual malformations, mainly involving loss of limbs ^1^; (ii) Minamata Disease resulting from pollution of seafood with excessive amounts of methylmercury and cerebral palsy in offspring of exposed pregnant women ^2^; and (iii) the increased prevalence of deafness and blindness following antenatal exposure to rubella ^3^. The associations with thalidomide, mercury and rubella were all discovered because the outcomes were so unusual.

One reason for failure to spot environmental features that could have initiated adverse consequences relates to the fact that often the woman is unaware that she is pregnant in the early weeks. Importantly, at this time the developing embryo is most vulnerable to adverse effects ^4^ – whether this is of a maternal infection, a drug ingested, a binge of alcohol or a traumatic event. Nevertheless, such exposures operating later in pregnancy can also have different but important consequences, particularly on the development of the brain ^5^.

There has been increasing awareness of the fact that exposures resulting in common adverse outcomes would be difficult to spot, unless one used a study that collected information prospectively during pregnancy and followed the offspring during childhood, adolescence and into adulthood. The aim of this paper is to describe the structure of the data collection used by the Avon Longitudinal Study of Parents and Children (ALSPAC), a population based, pregnancy cohort that collected data during pregnancy. We then aim to describe the problems which arose in order to inform a) future studies and b) all researchers using the pregnancy-based data from ALSPAC.

## Methods

### Study overview

ALSPAC was designed to assess the ways in which the environment interacts with the genotype to influence health and development ^6– 8^. Pregnant women resident in the study area in south-west England with an expected date of delivery between 1 ^st^ April 1991 and 31 ^st^ December 1992, were invited to take part. About 80% of the eligible population did so. The initial ALSPAC sample consisted of 14,541 pregnancies; of these initial pregnancies, there was a total of 14,676 foetuses, resulting in 14,062 live births and 13,988 children who were alive at one year of age. Information on the cohort parents and their offspring was collected using a variety of methodologies including self-completion questionnaires sent to study women, their partners, teachers, and from the age of five, the study child. The study also used direct examination under standardised conditions and linkage to educational data from the school system and other administrative records. Please note that the study website contains details of all the data that is available through a fully searchable data dictionary and variable search tool ( http://www.bristol.ac.uk/alspac/researchers/our-data/).

Ethical approval for the study was obtained from the ALSPAC Ethics and Law Committee (ALEC; IRB00003312) and the Local Research Ethics Committees ^9^. Detailed information on the ways in which confidentiality of the cohort is maintained may be found on the study website: http://www.bristol.ac.uk/alspac/researchers/research-ethics/. Informed consent for the use of data collected via questionnaires and clinics was obtained from participants following the recommendations of the ALSPAC Ethics and Law Committee at the time.

### Recruitment

The study was designed to identify all eligible women based on (a) their area of residence (the former county of Avon); and (b) their expected date of delivery (April 1991 to December 1992 inclusive). The study area encompassed the city of Bristol and its surrounds, which includes the large coastal town of Weston-super-Mare, 27 miles from Bristol, and a mixture of rural and semi-urban areas. At the time a variety of prenatal health systems were in place within the area. Those pregnancies deemed to be at low risk were primarily managed by general practitioners in their local general practices. Consultant obstetricians were based at three hospitals (two in Bristol and one in Weston-super-Mare). High-risk women were transferred for antenatal care to the Bristol hospitals where the neonatal baby units were available if required. Thus, there were a number of different local health services in the area that may have had contact with eligible women at different gestations. It was therefore decided to use a variety of strategies to contact the women (both using the health service but also encouraging women to self-refer); this methodology is reflected in the wide range of gestations at which the women had first contact with the study.

**Figure 1.**
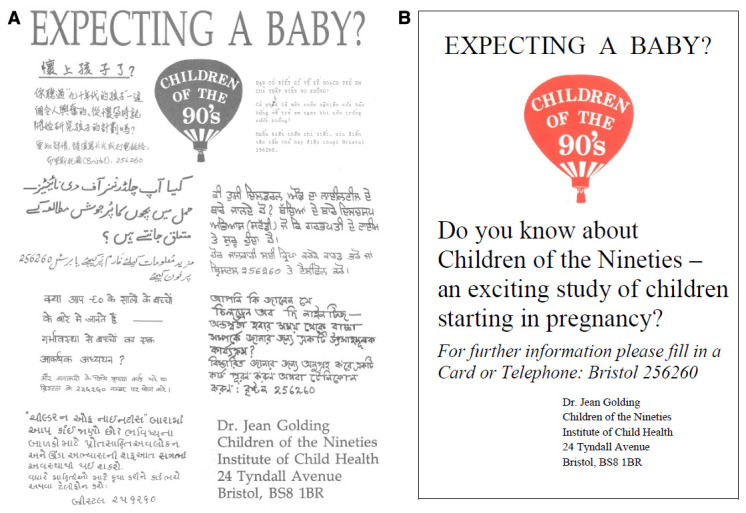
ALSPAC recruitment poster (multi-lingual) and the English language translation.

Posters were displayed in a variety of different places - including pharmacies, libraries, mother and toddler groups, pre-school playgroups, general practitioner waiting-rooms, antenatal clinics and any other area where a woman in early pregnancy was likely to visit. A multi-language version was also produced in association with “Maternity Links” (a local support agency for non-English speaking women). The poster displayed the logo of the study ‘Children of the Nineties’ and asked interested pregnant women to get in touch with the study team ( Figure 1). In addition, there was considerable local and national coverage in the press, radio and television.

Local community midwives discussed the study with pregnant women on their first visit and gave them a postcard to send off for further details. These cards were produced in a variety of different languages reflecting the ethnic groups common to Avon at that time (Vietnamese, Urdu, Bengali, Punjabi, Chinese, Hindi and Gujarati). Women completed their cards with their full names and dates of birth, addresses, the dates of their last menstrual period (LMP) and expected dates of delivery and returned them (in Freepost envelopes) to the study office.

Once the card had been received at the study office, a brochure was sent to the woman. This outlined the reason for carrying out the study and explained that the women themselves would not benefit tangibly, but that the major benefits were likely to be for the next generation. It informed the woman that there was no compulsion for her to take part, and that even if she enrolled in the study, she was free to opt out at any point. Thirdly, it emphasised the confidential nature of the information that would be collected and promised that at no time would the names of the woman and/or child be linked to the confidential information collected. Fourthly, it explained that biological samples would be taken, but that these would not be analysed without her signed consent, and finally it stated that the information given would also be linked to information from medical records unless she let us know that she did not want us to do this. The woman was also informed in this brochure that we would assume that she wanted to participate in the study unless she informed us otherwise. There were no exclusion criteria, and women were encouraged to enrol as early in pregnancy as possible.

### Questionnaire administration during pregnancy

Approximately seven days after the brochure had been sent out and, provided we had not heard from the woman that she wanted to opt out, the first questionnaire was posted to her. The nature of the questionnaire depended on her gestation at enrolment ( Table 1). In brief, there were a total of four questionnaires administered to the woman during pregnancy; two were scheduled to be sent at fixed time points:

(B Files) ‘Having a Baby’ at 18 weeks’ gestation

(C Files) ‘Your Pregnancy’ at 32 weeks’ gestation.

**Table 1.** The instructions as to when the different questionnaires were to be sent out according to the gestation at enrolment and the name of the questionnaire.

Gestation at enrolment (weeks)	ENV	HAB	YP *	AY	YHL
<11	<11	18	32	14	-
11–14	11–14	18	32	23	-
15–18	22	18	32	26	-
19	24	19	32	28	-
20	24	20	32	28	-
21	24	21	32	28	-
22	28	22	32	36	-
23	28	23	32	36	-
24	-	-	29	33	24
25	-	-	30	34	25
26	-	-	31	35	26
27	-	-	32	36	27
28	-	-	32	36	28
29	-	-	32	36	29
30	-	-	33	37	30
31	-	-	31	X	34
32	-	-	32	X	35
33	-	-	33	X	36
34	-	-	34	X	37
35	-	-	35	X	37
36	-	-	36	X	38
37	-	-	37	X	39
38	-	-	38	X	40
39	-	-	39	X	41
40	-	-	40	X	41

X = administered four months post-delivery; ENV = Your Environment; AY= About Yourself; HAB= Having a Baby; YP= Your Pregnancy; YHL= Your Home and Lifestyle. *For women enrolled after 40 weeks, a short questionnaire ‘Filling the Gaps’ was sent 12 months post-delivery.

Provided the woman enrolled before 14 weeks’ gestation, the third questionnaire (A Files) ‘Your Environment’ was sent to her immediately after enrolment. This questionnaire was designed in particular to identify those features of the early environment that might be responsible for detrimental effects on the foetus.

The fourth questionnaire (D Files) ‘About Yourself’ was mainly concerned with the woman’s past medical, social and environmental history, and consequently the time during pregnancy at which this was administered was relatively unimportant (majority received in the range 14–37 weeks). If necessary, therefore, this questionnaire was sent out postnatally.

For questionnaires administered during pregnancy the reminder and follow-up phase was fairly intensive. If a response had not been received within seven days, a reminder letter was sent. If the questionnaire had still not been received after a further 10 days, a second reminder letter was sent. Finally, if no response had been received after one month, a member of the study team either telephoned the woman or visited the home, and encouraged, or assisted, them in completing the questionnaire.

For women who did not enrol until six weeks after the 18-week contact, the ‘Having a Baby’ questionnaire was not likely to be valid. Much of the detail in that questionnaire was concerned with attitudes, activities and emotional well-being at that *particular point* in pregnancy. Nevertheless, for those women who had enrolled late, there was a certain amount of valid information concerning the environment and lifestyle that could be collected. For these women, therefore, the appropriate information, otherwise obtained from the questionnaires ‘Your Environment’ and ‘Having a Baby’, was combined into a single questionnaire ‘Your Home & Lifestyle’.

For a variety of reasons (including very preterm delivery) some women did not receive the questionnaire ‘Your Pregnancy’ which included questions on ethnic origin, educational, social, and occupational levels. It also included questions on early sexual experiences. All these questions that were not specific to the third trimester of pregnancy were therefore included in a short questionnaire entitled ‘Filling the Gaps’, which was administered 12 months post-delivery.

Despite rigorous piloting, the early questionnaires were found to have occasional errors that were corrected on future versions. These were identified by date of printing. In addition, there were two groups of women who had certain changes to their questionnaires, as described below.

### Second pregnancies

The women undergoing second pregnancies within the study recruitment period had a version of the questionnaire that omitted questions that would not have changed in a second pregnancy (e.g. ethnic origin). The editing process therefore copied over the answers to the first questionnaire sent to these women. The numbers answering the shortened A, C and D files were 100, 105 and 117 respectively.

### Women <16 years

The questionnaire ‘Your Pregnancy’ included intimate questions on sexual experiences. It was decided by the Ethics Committee that these were inappropriate for a girl under the legal age of consent, and so the questionnaire was adapted to omit these questions for the 30 girls concerned.

## Dataset

### The released data files

The data from the six questionnaires sent to the woman as described above were combined into four research data files as follows:

**A File** is predominantly the questions in ‘Your Environment’, but also includes a few relevant questions from ‘Your Health and Lifestyle’. The version of the questionnaire used is described in variable *a001*, and the gestation at which it was completed (based on the date of LMP and date of completion in completed weeks) in *a902*.

**B File** comprises questions from ‘Having a Baby’ and ‘Your Health and Lifestyle’. The version of the questionnaire used is described in variable *b001*, and the gestation at which it was completed (based on the date of LMP and date of completion in completed weeks) in *b924*.

**C File** consists of data from the questionnaires ‘Your Pregnancy’ and ‘Filling the Gaps’. The version of the questionnaire used is described in variable *c001*, and the gestation at which it was completed (based on the date of LMP and date of completion in completed weeks) in *c991*.

**D File** provides data from the questionnaire ‘About Yourself’. The version of the questionnaire used is described in variable *d001*, and the gestation (or time after delivery) at which it was completed (based on the date of LMP and date of completion in completed weeks) in *d990* and *d991*.

### The actual gestations at completion

In Table 2, we show the numbers of questionnaires completed by week since the LMP. These indicate the following peak times at completion:

A files: 45% at <15 weeks; 32% at 22–25 weeks

B files: 79% at 18–21 weeks

C files: 85% at 31–34 weeks

D files: 89% during pregnancy; 11% post-delivery

**Table 2.** Frequency of completion at each completed week of gestational age for each questionnaire.

Gestation at receipt (in weeks)	A	B	C	D
<7	152	5	0	5
7	195	0	0	2
8	319	0	0	0
9	559	6	1	5
10	774	4	0	6
11	889	1	1	1
12	1071	1	0	5
13	1049	14	1	9
14	1005	30	1	1203
15	577	16	0	411
16	201	28	2	253
17	130	46	1	136
18	85	5351	1	81
19	51	2407	4	62
20	31	1589	0	49
21	33	991	2	31
22	1935	591	6	42
23	631	369	11	2162
24	1253	244	16	757
25	444	167	26	364
26	256	141	32	236
27	199	126	65	1382
28	297	92	155	669
29	145	90	155	888
30	105	66	161	489
31	76	53	815	222
32	50	34	5854	160
33	29	13	2454	79
34	32	25	876	44
35	44	45	442	34
36	51	47	256	579
37	66	65	197	195
38	44	39	98	61
39	47	38	57	24
40	35	31	33	12
41	31	30	18	6
42	37	33	4	1
43	41	45	3	0
Post-delivery	288	279	562	1417
Total returned with known dates	13,257	13,274	12,310	13,888
Mode	22 weeks	18 weeks	32 weeks	23 weeks
Median	15 weeks	18 weeks	32 weeks	24 weeks

**A File**: ‘Your Environment’ plus some relevant questions from ‘Your Health and Lifestyle’; **B File:** ‘Having a Baby’ and ‘Your Health and Lifestyle’; **C File:** ‘Your Pregnancy’ and ‘Filling the Gaps’; **D File:** ‘About Yourself’.

Thus, the aim of obtaining data at around 18 and 32 weeks was fairly successful, with almost 80% of B files and 85% of C files completed during a four-week period. The aim to get the A file data within the first trimester, however, was successful for less than half the pregnancies. As planned, the D files were completed at various gestations throughout pregnancy and the postnatal period.

### How to refer to the gestations at which the data were obtained

Given the above distributions, we suggest that the following descriptions are used for each data set:

A files – 45% 1 ^st^ trimester

B files – 18–21 weeks

C files – 31–34 weeks

D files – 89% during pregnancy

It should be noted that although labelled ‘gestation’, the variables *a902, b924, c991, d990, pb900* and *pa900* denote the number of weeks after the woman’s stated LMP. No correction has been made for the final estimate of gestation as obtained from the clinical records. In addition, these variables include women who completed questionnaires after the baby was delivered but different variables describe the age of the child at completion in these cases.

## Conclusions

### A note for users of the data

The Avon Longitudinal Study of Parents and Children (ALSPAC) was designed with the aim of determining features of the environment that may influence the health and development of the baby through childhood and into adulthood ^5^. Knowing that the embryo is susceptible to early insults (whether from drugs, infections or other impacts), the aim was to start the study as early in pregnancy as possible, and to collect as much relevant information as possible throughout pregnancy. As can be seen above, we were not very successful in obtaining the environmental data in the first trimester. This needs to be taken into account when using data from the A files and where necessary data returned at appropriate gestations can be selected.

## Ethical approval and consent

Ethical approval for the study was obtained from the ALSPAC Ethics and Law Committee and the Local Research Ethics Committees. Informed consent for the use of data collected via questionnaires and clinics was obtained from participants following the recommendations of the ALSPAC Ethics and Law Committee at the time. Children were invited to give assent where appropriate. Study participants have the right to withdraw their consent for elements of the study or from the study entirely at any time. Full details of the ALSPAC consent procedures are available on the study website ( http://www.bristol.ac.uk/alspac/researchers/research-ethics/).

## Data availability

ALSPAC data access is through a system of managed open access. The steps below highlight how to apply for access to the data included in this data note and all other ALSPAC data:

1. Please read the ALSPAC access policy ( http://www.bristol.ac.uk/media-library/sites/alspac/documents/researchers/data-access/ALSPAC_Access_Policy.pdf) which describes the process of accessing the data and samples in detail, and outlines the costs associated with doing so.

2. You may also find it useful to browse our fully searchable research proposals database ( https://proposals.epi.bristol.ac.uk/?q=proposalSummaries), which lists all research projects that have been approved since April 2011.

3. Please submit your research proposal ( https://proposals.epi.bristol.ac.uk/) for consideration by the ALSPAC Executive Committee. You will receive a response within 10 working days to advise you whether your proposal has been approved.
