# Supplementary material for: Gestation at completion of prenatal questionnaires in ALSPAC
Source: Wellcome Open Res. 2020 Oct 7;5:100. [Version 1] doi: 10.12688/wellcomeopenres.15976.2 (PMC7569746; doi:10.12688/wellcomeopenres.15976.2)
Supplement: Peer review history

# Peer review: Reviewer response for version 2

**DOI:** 10.21956/wellcomeopenres.17982.r40751

**Published:** 2020-10-16

**Authors:** Stephanie M. Eick

**Affiliations:** 1 Program on Reproductive Health and the Environment, Department of Obstetrics, Gynecology and Reproductive Sciences, University of California, San Francisco, San Francisco, CA, USA

The authors have fully addressed my concerns.

Are sufficient details of methods and materials provided to allow replication by others?

No

Is the rationale for creating the dataset(s) clearly described?

Yes

Are the datasets clearly presented in a useable and accessible format?

Yes

Are the protocols appropriate and is the work technically sound?

Yes

Reviewer Expertise:

epidemiology, environmental health, reproductive health

I confirm that I have read this submission and believe that I have an appropriate level of expertise to confirm that it is of an acceptable scientific standard.

# Peer review: Reviewer response for version 1

**DOI:** 10.21956/wellcomeopenres.17521.r40219

**Published:** 2020-09-09

**Authors:** Stephanie M. Eick

**Affiliations:** 1 Program on Reproductive Health and the Environment, Department of Obstetrics, Gynecology and Reproductive Sciences, University of California, San Francisco, San Francisco, CA, USA

In this manuscript, the authors provide a detailed overview of the ALSPAC study. There is detailed information about study recruitment and the timing of when certain exposures were measured. This manuscript is well-written and would be relevant for researchers who are interested in using this data. I have several specific comments outlined below that I think would improve the clarity of this manuscript.

- Abstract: The sentence “The Avon Longitudinal Study of Parents and Children (ALSPAC) is a prime example of such a cohort” – what type of cohort, a pregnancy cohort? A well-constructed cohort? I think this should be clarified.
- First sentence in the second paragraph of the introduction: the word “caused” seems too strong here, I recommend softening it. Very few things have a single definitive “cause” in epidemiology and nearly everything is multi factorial.
- Second paragraph in the introduction needs citations. There are a lot of claims made with no reference to support them.
- Study population: What gestational ages were women targeted for recruitment? The authors state that women were recruited throughout pregnancy but was one window preferred and what was the earliest time point that women were eligible for the study?
- Recruitment: It would be helpful to specify a little more what was a “low risk” pregnancy and define more specifically the inclusion and exclusion criteria. Was there an age range for mothers, language considerations, anything beyond specific geographic region, etc.?
- Table 1: This table isn’t very clear to me as currently presented. The columns “ENV” to “YHL”, are these the different gestational age ranges that the surveys were sent out at? The heading should be more detailed here or maybe this information would be better represented as a figure.
- Table 2: To me, this table is really portraying much information as currently presented – I think it would be helpful if this information was presented as N (%) instead of just N. For example, what is the total? The raw numbers aren’t very intuitive to me without some form of reference. I think it would also be helpful if this information was grouped by gestational age as opposed to separating out each individual week gestation. My instinct is that if they were grouped somehow (maybe by 4 weeks?) there likely wouldn’t be that much difference between the individual weeks – although this is hard to tell because as presented the raw N isn’t very informative. It would also be helpful to clarify if the gestational ages in this table indicate that the questionnaire was sent to the ppt or the gestational age that the ppt actually filled it out – I imagine that could lead to some misclassification if women didn’t fill out the survey right away. I’m not sure reporting the mode is necessary, my thought would be to report the mean for consistency with other studies. Lastly, what % of ppts were lost to follow up at each time point? There is a row for “total returned” but I think it would be more informative to know what % of the total possible sample that was.
- What % of women had a second pregnancy during the study time frame and were enrolled in the study with that second pregnancy?
- For readers who are interested in using this data and are reading this manuscript as a general description of the study, I think it would be helpful to provide some general demographics as a descriptor of the overall cohort.

Are sufficient details of methods and materials provided to allow replication by others?

No

Is the rationale for creating the dataset(s) clearly described?

Yes

Are the datasets clearly presented in a useable and accessible format?

Yes

Are the protocols appropriate and is the work technically sound?

Yes

Reviewer Expertise:

epidemiology, environmental health, reproductive health

I confirm that I have read this submission and believe that I have an appropriate level of expertise to confirm that it is of an acceptable scientific standard, however I have significant reservations, as outlined above.

# Response

**Published:** 2020-09-29

**Authors:** Jean Golding

**Affiliations:** University of Bristol, UK

Many thanks for your useful comments. I have answered them below, and changed the paper where indicated.

1. The Abstract: the words ‘of such a cohort’ have been changed to: ‘of a cohort where certain data were collected at specific time points and others at variable times depending on the gestation at enrolment’.

2. The word ‘caused’ has been changed to ‘initiated’.

3. The statement in this paragraph is now accompanied by a reference to a comprehensive review (new ref 4).

4. The following sentence has been added to clarify. ‘There were no exclusion criteria, and women were encouraged to enrol as early in pregnancy as possible.’

5. As stated in the sentence added above, there were no exclusions, and all pregnant women were targeted. ‘Low risk’ was mentioned as a description of where such pregnant women were likely to receive their obstetric care – but did not change the way in which the pregnant woman was approached.

6. Apologies for omitting the meaning of the questionnaires PQ and YYE. These were questions sent to the partner of the pregnant woman, and have now been omitted, as they will be the topic of a separate Data Note.

7. (a) Table 2 presents the numbers of women responding to the various questionnaires, with the actual gestation at completion. This table is presenting the details so that a researcher can determine the numbers that would be available for any particular analysis based on the gestational window of interest; for this reason we feel it is important to display the data by actual week of gestation. The interested reader can calculate percentages from the totals at the foot of each column.

(b)  We have quoted the median and mode gestational ages. We have not quoted the mean as the distributions are so skewed for two of the variables that the mean would be inappropriate.

(c)  The referee asks for % lost to follow-up. This is not as obvious a question as it appears. As the referee will be aware, a survey of pregnancy is a fluid event, with people enrolling, gestations changing as more information becomes available, pregnancies miscarrying or being terminated, and delivering preterm. Consequently the numbers eligible will vary by week of gestation as well as by week of enrolment. Calculation of this statistic is too detailed for this simple data note.

8. The number of second pregnancies completing the AY questionnaire was 117. This information has been added to the appropriate section, as has the number of women who had shorter questionnaires because they were < 16 years of age.

9. The reviewer requests overall demographics of the study. We have not included this as it is adequately covered in the references quoted [refs 6-8].

# Peer review: Reviewer response for version 1

**DOI:** 10.21956/wellcomeopenres.17521.r38815

**Published:** 2020-06-05

**Authors:** Lucy Pembrey

**Affiliations:** 1 Department of Medical Statistics, London School of Hygiene and Tropical Medicine, London, UK

This Data Note describes how data were collected with questionnaires at several points during pregnancy from mothers enrolled in the Avon Longitudinal Study of Parents and Children (ALSPAC) from April 1991 to December 1992. Details of the recruitment procedures are provided with a detailed description of the timing of administration of the six different questionnaires according to gestational age at the time of enrolment. The four available datasets are described, and the number of questionnaires collected at each gestational age for each dataset are shown.

 Given the need to enrol women at different stages of pregnancy and the importance of taking into account the gestational age at the time questions were asked for certain environmental exposures, this Data Note provides a useful summary of the complexity of the ALSPAC pregnancy questionnaires. ALSPAC data are now widely used by researchers in the UK and internationally, accessed via application to the ALSPAC Executive Committee. Researchers using variables from the pregnancy questionnaires can be referred to this publication and it can be referenced in papers of their results.

 I have a few minor comments as follows:

1. The last sentence of the Introduction could be re-phrased for clarity, from ‘Then to describe the problems arising in order to inform…’ to ‘We then aim to describe the problems which arose in order to inform…’ 
2. In Table 1, please indicate in the footnote what PQ in column 3 and YYE in column 5 refer to. I can’t see where these are mentioned in the text. If these are additional questionnaires, further explanation in the text on page 4 may be needed.
3. The first sentence on page 5 could be re-phrased for clarity, from ‘Nevertheless, for those women who had enrolled late, there was a certain amount of information concerning the environment and lifestyle that could and should validly be collected.’ to ‘Nevertheless, for those women who had enrolled late, there was a certain amount of valid information concerning the environment and lifestyle that could be collected.’

Are sufficient details of methods and materials provided to allow replication by others?

Yes

Is the rationale for creating the dataset(s) clearly described?

Yes

Are the datasets clearly presented in a useable and accessible format?

Yes

Are the protocols appropriate and is the work technically sound?

Yes

Reviewer Expertise:

epidemiology, birth cohort studies

I confirm that I have read this submission and believe that I have an appropriate level of expertise to confirm that it is of an acceptable scientific standard.

# Response

**Published:** 2020-09-29

**Authors:** Jean Golding

**Affiliations:** University of Bristol, UK

Thank you so much for your comments. We have changed the text in the way you have suggested in points 1 and 3. In regard to point 2 we have omitted the labels in the Table. They referred to questionnaires to the partners which are not the topic of this questionnaire.
